# Automated Multireplicate Quantification of Persistent HIV-1 Viremia in Individuals on Antiretroviral Therapy

**DOI:** 10.1128/JCM.01442-20

**Published:** 2020-11-18

**Authors:** Jana L. Jacobs, Melissa A. Tosiano, Dianna L. Koontz, Brittany Staines, Andrew Worlock, Karen Harrington, Sonia Bakkour, Mars Stone, Kathleen Shutt, Michael P. Busch, John W. Mellors

**Affiliations:** a Division of Infectious Diseases, Department of Medicine, University of Pittsburgh School of Medicine, Pittsburgh, Pennsylvania, USA; b Hologic Incorporated, San Diego, California, USA; c Department of Laboratory Medicine, University of California San Francisco, San Francisco, California, USA; d Vitalant Research Institute, San Francisco, California, USA; Rhode Island Hospital

**Keywords:** ART, HIV-1, antiretroviral therapy, viremia

## Abstract

Clearance of low-level viremia that persists in most HIV-1-positive individuals on antiretroviral therapy (ART) is an important milestone for efforts to cure HIV-1 infection. The level of persistent viremia on ART is generally below the lower limit of quantification (LOQ) of current FDA-cleared plasma HIV-1 RNA assays (20 to 40 copies/ml) but can be quantified by reverse transcriptase PCR (RT-PCR) assays with single-copy sensitivity. Such assays require multistep manual methods, and their low throughput limits the capacity to monitor the effects of interventions on persistent viremia. Recently, S. Bakkour, X. Deng, P. Bacchetti, E. Grebe, et al. (J Clin Microbiol 58:e01400-20, 2020, https://doi.org/10.1128/JCM.01400-20), reported the use of multiple replicates and Poisson statistics to infer HIV-1 RNA concentrations below the commercial LOQ of an automated platform (Hologic Panther Aptima). Here, we evaluate the detection and quantitation of low-level viremia using the following two adaptions of the automated platform: a multireplicate strategy (9×) and a concentrated single-replicate strategy in which 5 ml of plasma is concentrated by centrifugation (1×, concentrated). We compare these new methods to a recently reported manual *integrase*-targeting single-copy assay version 2 (iSCA v2). Using laboratory-generated HIV-1 RNA plasma samples at known concentrations, all three methods had similar sensitivity for HIV-1 RNA detection, although iSCA v2 was most sensitive (95% LOD, 2.3 copies/ml), 9× was marginally less sensitive (95% LOD, 3.0 copies/ml), and 1×, concentrated was least sensitive (95% LOD, 3.9 copies/ml). In contrast, for clinical plasma samples, 9× had greater sensitivity than iSCA v2 (82% of samples were quantifiable compared with 62% of samples by iSCA v2). These results support 9× as an acceptable high-throughput alternative to iSCA v2 for quantifying low-level viremia in individuals on ART.

## Abstract (precis)

Clearance of low-level viremia that persists in most HIV-1-positive individuals on antiretroviral therapy (ART) is an important milestone for efforts to cure HIV-1 infection. The level of persistent viremia on ART is generally below the lower limit of quantification (LOQ) of current FDA-cleared plasma HIV-1 RNA assays (20 to 40 copies/ml) but can be quantified by reverse transcriptase PCR (RT-PCR) assays with single-copy sensitivity. Such assays require multistep manual methods, and their low throughput limits the capacity to monitor the effects of interventions on persistent viremia.

## INTRODUCTION

Current antiretroviral therapy (ART) produces logarithmic decreases in plasma viremia, eventually reaching levels below the lower limit of quantification (LOQ) for FDA-cleared HIV-1 RNA assays of 20 to 40 copies/ml (1). Assays developed to detect single copies of HIV-1 RNA in plasma revealed that the majority of individuals on clinically effective ART have persistent, low-level viremia (1 to 3 copies/ml) attributed to a long-lived reservoir of HIV-1-infected cells (2–14). Single-copy assays (SCAs) have become important in clinical trials of experimental interventions targeting HIV-1 reservoirs to assess their impact on persistent viremia. SCAs involve extensive centrifugation to pellet HIV-1 virions and cumbersome manual nucleic acid extraction procedures, followed by steps to reverse transcribe extracted viral RNA to DNA for detection by real-time PCR. The extensive hands-on time required limits throughput and increases the chance for human error and variation in results. Because of these issues, adaptation of an automated platform for the quantitation of low-level viremia below 20 to 40 copies/ml is imperative.

It has been observed that results reported by either Roche or Abbott automated platforms as <20 or <40 copies/ml, respectively (also known as “detected but not quantifiable”), are almost always detected and quantified by a manual single-copy assay, whereas automated platform results indicating “no target detected” are associated with a significantly lower frequency of HIV-1 RNA detection by manual single-copy assays (15, 16), indicating that detection of HIV-1 RNA below the limit of detection (LOD) of the commercial assays correlate with levels of HIV-1 RNA detected by a manual single-copy assay. This finding suggests that automated platforms may provide useful information for monitoring low levels of HIV-1 viremia in intervention studies, even below the approved LOD. Indeed, some automated platforms have been investigated for their use in monitoring HIV-1 plasma viremia below the approved LOQ. For example, recent studies employed a multiple replicate strategy on an automated platform using a signal/cutoff ratio method for readout below the published LOD to monitor low levels of HIV-1 (17, 18).

In the interest of adapting automated platforms to quantify HIV-1 RNA below the published LOD, Bakkour et al. have recently reported the use of the Aptima HIV-1 Quant test for the quantitation of HIV-1 RNA in plasma below the published limit of quantitation (LOQ; 30 copies [cps]/ml) on the Hologic Panther platform using a multiple replicate strategy (19, 20). They designed a hybrid algorithm wherein values are assigned using Poisson modeling of a binomial output for those with at least 1 negative (below LOD) replicate of 9, 18, or 45 total replicates per sample; those with no negative replicates whose quantities are below the LOQ are assigned a value using a proprietary algorithm.

Here, we describe a further assessment of the automated multiple replicate strategy (9×) by comparing results obtained using 9 replicates (5-ml total) from plasma samples spiked with known concentrations of HIV-1 to results obtained from the same plasma volume using the recently published manual *integrase*-targeting single-copy assay version 2 (iSCA v2) with a 95% LOD of ∼1 cp/ml in a 5-ml sample (21). We chose 5 ml of plasma because this is a reasonable volume to be obtained from participants in clinical trials. Assay performance was further compared using plasma samples from HIV-1-positive donors on ART. We also assessed an alternate centrifuge-based method for adapting the Panther platform to quantify HIV-1 in plasma below the published LOQ, in which 5-ml plasma was concentrated by centrifugation to 0.7 ml and quantified in one replicate (1×, concentrated).

## MATERIALS AND METHODS

### Clinical specimens.

All participants who donated plasma were volunteers at the University of Pittsburgh AIDS Clinical Trials Unit and provided written informed consent. Sample collection was approved by the University of Pittsburgh Institutional Review Board. Plasma specimens were collected using EDTA tubes from individuals on effective ART (plasma HIV-1 RNA, <20 copies/ml by Roche COBAS AmpliPrep/COBAS TaqMan HIV-1 test) for at least 4 years. Participants underwent phlebotomy (100 ml to 180 ml), and blood was processed within 4 hours of collection. Plasma was separated by centrifugation of whole blood at 400 × *g* for 10 minutes, and then plasma was centrifuged at 1,350 × *g* for 15 minutes. For both centrifugations, a Thermo Scientific Sorvall Legend X1 centrifuge accommodating 50-ml tubes was used. The cell-free plasma was harvested and stored at −80°C in 1.5-ml aliquots. All plasma samples were collected between 2013 and 2019.

### Hologic Panther methods.

**FIG 1:**
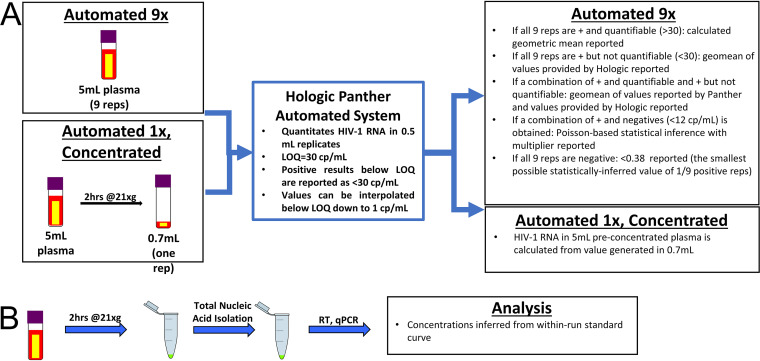
Workflow and analysis of automated 9× and automated 1×, concentrated (A) and iSCA v2 (B). Three methods to quantify low-level HIV-1 RNA in plasma of HIV-1-positive but virologically suppressed individuals were compared. Two methods use the Hologic Panther Aptima automated platform (A) and the third uses a published manual single-copy assay (iSCA v2) (B).

All methods using the Hologic Panther platform were performed using the Aptima HIV-1 Quant assay kit following the manufacturer’s instructions. All samples run using the Aptima kit contained an internal positive control. Samples were centrifuged in a Thermo Scientific Sorvall Legend X1 centrifuge accommodating the Hologic Panther Specimen Aliquot Tubes prior to being loaded on the Hologic Panther instrument as directed. 9× quantifies HIV-1 RNA in 5 ml of plasma by individually processing 9 0.5-ml replicates (reps) (Fig. 1A). Depending on the results of the 9 independent replicates, 1 of the following 5 analyses is used: (i) if all 9 reps are positive and quantifiable (>LOQ of 30 cps/ml), then the geometric mean of the values for the 9 reps is reported; and (ii) if all 9 reps are positive but not quantifiable (>LOD of 12 cps/ml but <LOQ of 30 cps/ml), values were provided by Hologic from the instrument logs through proprietary software. The algorithm used in the software can calculate results below 30 copies/ml using the data generated during the runs, but this information is not reported to the user during routine testing. Results below 30 copies/ml are for research use only and are not for use in diagnostic procedures. The geometric mean of these values was reported (iii) if a combination of positive and quantifiable (>LOQ of 30 cps/ml) and positive but not quantifiable (>LOD of 12 cps/ml but <LOQ of 30 cps/ml) replicates is observed, the geometric mean of the quantifiable values and the Hologic-provided values (as in analysis ii above) are reported; (iv) if a combination of positive and negative replicates (<LOD of 12 cps/ml) is observed among the 9 replicates, then a combinatorial method of Poisson modeling with a multiplier developed by Bakkour et al. is used to assign values; and (v) if all 9 reps are negative (<LOD of 12 cps/ml), then a value of <0.38 cps/ml is reported (0.38 cps/ml is the theoretical limit of detection, which is the lowest possible Poisson value of 1/9 positive replicates).

The 1×, concentrated quantifies HIV-1 RNA in 5-ml plasma by centrifuging at 21,000 × *g* for 2 h at 4°C in a refrigerated Eppendorf 5427R centrifuge with a rotor to accommodate 5-ml tubes. Supernatant is removed with care to avoid pellet disruption, volume is brought to 0.7 ml, and the whole 0.7-ml run as one replicate on the Panther platform. The concentration in the original 5 ml is calculated from the result from the concentrated 0.7-ml sample (Fig. 1A).

### *Integrase*-targeting single-copy assay version 2.

iSCA v2 was performed as described with no modifications (21). Briefly, plasma was cleared of debris by a brief centrifugation, and then virions were pelleted by refrigerated centrifugation at 21,000 × *g* for 2 h. Virions were directly lysed by addition of 3 M guanidinium hydrochloride (GuHCl) supplemented with 50 mM Tris-HCl, 1 mM CaCl_2_, and 1 mg/ml proteinase K and by incubation at 42°C for 1 h, followed by addition of 6 M guanidinium thiocyanate (GuSCN) supplemented with 50 mM Tris-HCl, 1 mM EDTA, and 600 μg/ml of glycogen. Total nucleic acids were then precipitated with 100% isopropanol, washed with 70% ethanol, and resuspended in 65 μl of 5 mM Tris-HCl supplemented with 1 mM dithiothreitol (DTT) and 1,000 units/ml RNasin. Reverse transcription (RT) followed by quantitative PCR (qPCR) targeting the HIV-1 *integrase* gene was performed on 50/65 μl of total nucleic acid, and quantification of HIV-1 RNA copies was inferred from an in-run standard curve (Fig. 1B).

### Statistical analysis.

Statistical analysis was performed using GraphPad Prism 8 and SAS 9.4. To assign estimates of plasma HIV-1 RNA using the Panther 9× for samples with a mixture of positive and negative replicates, a worksheet was provided by Bakkour et al. consisting of estimates for each possible outcome (e.g., 1/9 positive replicates and 2/9 positive replicates) derived from an online maximum likelihood estimation calculator (22) and adjusted by the calibration factor of 1.6 (determined by Bakkour et al. by calibration of the estimates of known standards [19]). To determine the 95% limit of detection (LOD) of each of the three methods, samples with detectable HIV-1 RNA were designated a “hit” and samples with undetectable HIV-1 RNA were designated a “nonhit.” LODs were calculated using maximum likelihood estimation SAS macro (LoD_MLE SAS macro) (23).

## RESULTS

The following three methods for quantifying low-level HIV-1 in plasma of clinically suppressed individuals were compared: (i) manual single-copy assay (iSCA v2), (ii) nine replicates on a Hologic Panther automated system (9×), and (iii) concentration by centrifugation prior to using a Hologic Panther automated system (1×, concentrated). A diagram detailing the three different methods and analyses are shown in Fig. 1, and further descriptions can be found in the Materials and Methods section.

**FIG 2:**
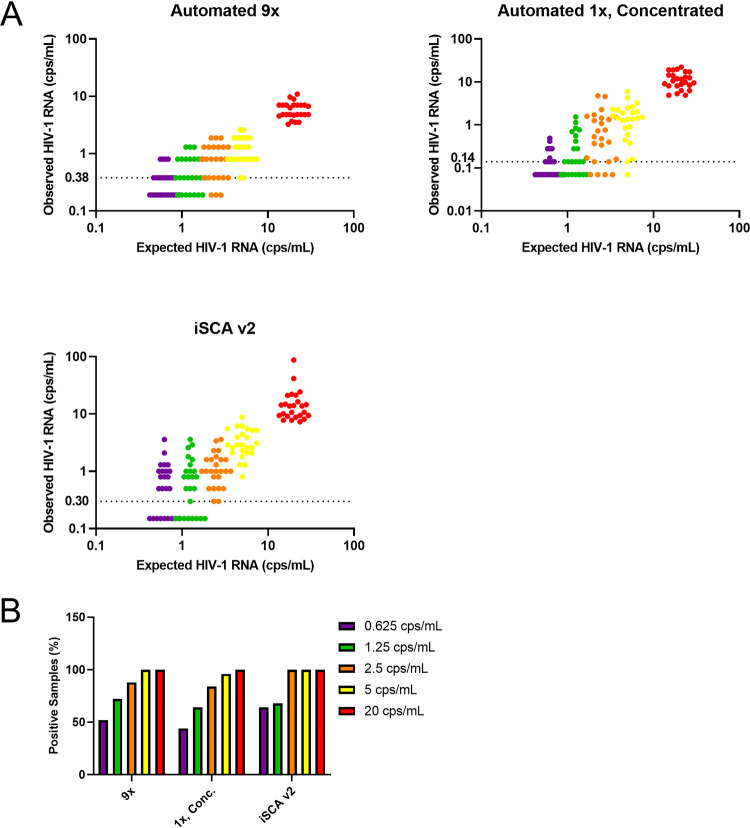
Results by assay type for 5 nominal HIV-1 RNA copy controls. (A) Standards from the Virology Quality Assurance laboratory at Rush University at 20 (red), 5 (yellow), 2.5 (orange), 1.25 (green), and 0.625 (purple) cps/ml were quantified by 9×, 1×, concentrated, and iSCA v2. For each of the methods, all 25 observed values are plotted for the 5 nominal copy standards. Samples that had no signal detected are plotted as half the theoretical limit of detection of 0.38 copies/ml for 9×, 0.14 copies/ml for 1×, concentrated, and 0.30 copies/ml for iSCA v2. (B) Percentage of samples with HIV-1 RNA detected at or above the limit of detection (% positive) is shown for each nominal concentration.

To begin the comparison of the three methods, we used standards obtained from the Viral Quality Assurance (VQA) laboratory at Rush University, which consist of known amounts of HIV-1 virions quantified by the Roche COBAS TaqMan HIV-1 test, that were diluted and spiked into HIV-1 negative plasma. For each of the three methods compared, we tested 5 ml of standards containing 20, 5, 2.5, 1.25, 0.625, and 0 cps/ml HIV-1. Five independent experiments consisting of 5 independent replicates were run for each concentration using each of the three methods, for a total of 25 experimental replicates. All three methods quantified VQA standards lower than the expected value, with iSCA v2 achieving values closest to expected (Fig. 2A and Table 1). The mean coefficient of variation (%CV) for 9× and 1×, concentrated were similar, at 33.6% and 35.8%, respectively (Table 1). The mean %CV for iSCA v2 was higher at 68.4%, likely reflecting the greater technical variation and hands-on time required to perform iSCA v2. The percentage of total experimental replicates detected was similar across methods, with all three methods achieving detection at the lowest concentration of 0.625 cps/ml, although iSCA v2 obtained the highest percent detection of 64% at 0.625 cps/ml (Fig. 2B and Table 2). The 1×, concentrated achieved the lowest percent detection of the three methods, falling to 96% detection at 5 cps/ml, while the other two methods maintained 100% detection at this concentration. A similar trend was evident when LOD_95_ (95% confidence interval) was determined for each method; iSCA v2 had the lowest LOD_95_ of 2.3 (1.6, 3.0) cps/ml, followed by 9× with LOD_95_ of 3.0 (2.1, 3.8) cps/ml, and 1×, concentrated with LOD_95_ of 3.9 (2.8, 5.0) cps/ml (Table 2). Importantly, none of the 25 reps of 0-cps/ml standards were detectable by any of the three methods (Table 2). Because of the reduced sensitivity and throughput of the 1×, concentrated compared with that of 9×, we decided not to move the 1×, concentrated into the next phase of evaluation.

**TABLE 1:** Summary of HIV-1 RNA quantification and variance by assay method

Method^*a*^	Expected concn (cps/ml)	Mean (± SD) observed concn (cps/ml)	%CV	Mean %CV
Automated 9×	20.0	6.08 ± 1.93	31.7	33.6
	5.0	1.27 ± 0.63	49.6	
	2.5	0.87 ± 0.31	35.6	
	1.2	0.55 ± 0.16	29.1	
	0.6	0.36 ± 0.08	22.2	
Automated 1×, concentrated	20.0	11.70 ± 2.56	21.9	35.8
	5.0	1.70 ± 0.58	34.1	
	2.5	1.04 ± 0.57	54.8	
	1.2	0.33 ± 0.13	39.4	
	0.6	0.14 ± 0.04	28.6	
iSCA v2	20.0	17.12 ± 16.40	95.8	68.4
	5.0	3.52 ± 1.92	54.5	
	2.5	1.30 ± 0.90	69.2	
	1.2	1.0 ± 0.60	60	
	0.6	0.80 ± 0.50	62.5	

a A total of 25 replicates were run for each concentration and method.

**TABLE 2:** Percent detection and estimated 95% and 50% LOD by assay method

Method	Expected concn (cps/ml)	No. detected	Total tests	Percent detection	LOD_95_ (95% CI) (cps/ml)	Estimated LOD_50_ (cps/ml)
Automated 9×	20.0	25	25	100	3.0 (2.1, 3.8)	<0.625
	5.0	25	25	100		
	2.5	22	25	88.0		
	1.2	18	25	72.0		
	0.6	13	25	52.0		
	0	0	25	0		
Automated 1×, concentrated	20.0	25	25	100.0	3.9 (2.8, 5)	0.625–1.25
	5.0	24	25	96.0		
	2.5	21	25	84.0		
	1.2	16	25	64.0		
	0.6	11	25	44.0		
	0	0	25	0		
iSCA v2	20.0	25	25	100	2.3 (1.6, 3)	<0.625
	5.0	25	25	100		
	2.5	25	25	100		
	1.2	17	25	68.0		
	0.6	16	25	64.0		
	0	0	25	0		

**FIG 3:**
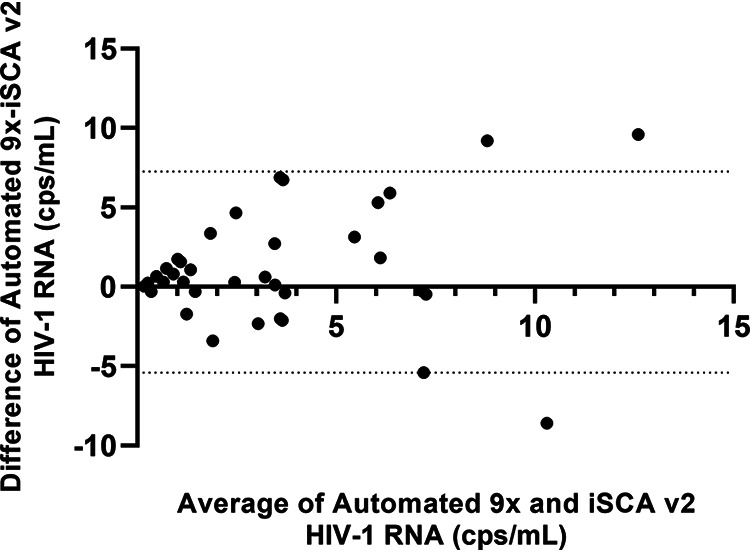
Bland Altman plot clinical plasma samples quantified by automated 9× and iSCA v2. Samples (*n* = 49) that quantified below the published LOQ of the Aptima assay (30 copies/ml) were plotted for Bland Altman analysis. Samples in which no HIV-1 RNA was detected are plotted as one-half of the theoretical limit of detection of 0.38 copies/ml for automated 9× and 0.30 copies/ml for iSCA v2. Dotted lines represent 95% confidence intervals.

We next evaluated the performance of 9× compared with iSCA v2 with 5 ml of plasma from 50 virologically suppressed donors on antiretroviral therapy. Overall quantification by 9× trended slightly higher than quantification by iSCA v2 according to Bland Altman analysis (Fig. 3). 9× was able to quantify 82% of the 50 samples tested, whereas iSCA v2 was able to quantify 62% of the 50 samples tested (one sample was omitted due to internal control failure), suggesting lower clinical sensitivity (see Table S1 in the supplemental material).

## DISCUSSION

Assessing changes in plasma HIV-1 RNA in response to treatment interventions has traditionally been achieved using either high-throughput automated platforms with published LOQs of 20 to 40 cps/ml or using manual single-copy assays. Since automated platforms provide incomplete information for assessing response to interventions in individuals with plasma HIV-1 RNA below the published LOQ and manual single-copy assays require significant technical expertise and time to complete, a high-throughput SCA is highly desirable. To this end, we have described here a further assessment of a multiple-replicate strategy on an automated platform for quantifying low-level HIV-1 viremia in plasma of virologically suppressed individuals, including performance evaluation compared with an optimized manual single-copy assay. The Aptima HIV-1 test run on the Panther Hologic is appropriate for this purpose since it is a high-throughput platform with random access sample loading. Additionally, since the amplification steps of the Aptima HIV-1 test rely on transcription-mediated amplification (TMA) rather than PCR, it is highly specific for HIV-1 RNA, precluding overestimation of HIV-1 RNA from detection of HIV-1 DNA that can occur with PCR-based automated platforms. Using 9× in place of the manufacturer’s recommended single-replicate protocol will allow a high-throughput assessment of changes in viremia ranging from <0.38 to 30 cps/ml in response to novel therapeutic interventions aimed at reversing HIV-1 latency and/or eliminating virus-producing cells toward the goal of achieving an ART-free remission of HIV-1 infection. Using 9× in place of iSCA v2 has the potential to boost weekly throughput by 5- to 10-fold.

Using laboratory-made standards, all three methods performed similarly, with iSCA v2 achieving both the greatest sensitivity and estimates of HIV-1 RNA that were closest to expected values. Importantly, for plasma samples from persons on suppressive ART, 9× tended to provide higher estimates of HIV-1 RNA than iSCA v2 and was able to detect a higher proportion of samples (82% of samples detected compared with 62% detected with iSCA v2). High variance in the quantitation of samples was observed, which is expected near the LOD of an assay, and in the case of single-copy detection, the expected variance was calculated by Poisson. Additionally, assay variance is dependent on both assay and sample characteristics. The performance of iSCA v2 with clinical samples may be affected by interfering substances concentrated during the initial centrifugation step, which is a step that is avoided during the automated HIV-1 kit magnetic bead-based extraction. The bead-based chemistry likely results in a cleaner nucleic acid extract. Multiple downstream steps of manual iSCA v2 are susceptible to these potential inhibitors, including nucleic acid resuspension after precipitation, reverse transcription, and PCR (24, 25). This likely explains the superior performance of iSCA v2 with lab-derived standards but not clinical samples. Nevertheless, manual single-copy assays will remain critical to use for plasma samples in cases where less than 5 ml of plasma is available. Indeed, iSCA v2 likely performs best with samples of limited volume because of lower concentrations of interfering substances following centrifugation.

Another important benefit of the automated method is the detection of two distinct targets within the HIV-1 genome and adequate detection of many different subtypes, including subtype C. Manual iSCA v2 primers are specific for only one target, and although the target is highly conserved, an alternative primer set is required to achieve adequate quantification of subtype C isolates. For samples of an unknown subtype, it would be beneficial to use the automated system to ensure detection and adequate quantification.

In conclusion, the automated 9× will likely be useful for high-throughput monitoring of low-level viremia in clinical trials involving individuals on ART. Further comparison, conducted in a blind manner, with iSCA v2 on samples is in progress, and evaluation of the performance of 9× with other sample types, such as cerebrospinal fluid, will provide additional insight.

## Supplementary Material

Supplemental file 1
