# Supplementary material for: Automated Multireplicate Quantification of Persistent HIV-1 Viremia in Individuals on Antiretroviral Therapy
Source: J Clin Microbiol. 2020 Nov 18;58(12):e01442-20. doi: 10.1128/JCM.01442-20 (PMC7685899; doi:10.1128/JCM.01442-20)
Supplement: Supplemental file 1 [file JCM.01442-20-s0001.pdf]

Table S1: Individual Results of Clinical Plasma Samples Tested by Automated 9x and iSCA v2

Donor	Automated 9x		iSCA v2 (cps/mL)	Donor	Automated 9x		iSCA v2 (cps/mL)
	Pos Reps[1]	cps/ mL			Pos Reps[1]	cps/ mL	
1	4/9	1.88	<0.3	26	2/9	0.80	<0.3
2	8/9	7.03	3.9	27	1/9	0.38	<0.3
3	4/9	1.88	0.3	28	9/9	4.5	9.9
4	5/9	2.59	4.6[2]	29	1/9	0.38	2.1
5	7/9	4.81	2.1	30	0/9	<0.38	<0.3
6	8/9	7.03	<0.3[3]	31	9/9	69	34.6
7	8/9	7.03	0.3	32	6/9	3.52	3.9
8	0/9	<0.38	0.5	33	8/9	7.03	5.2
9	0/9	<0.38	0.5	34	1/9	0.38	<0.3
10	0/9	<0.38	<0.3	35	0/9	<0.38	3.6
11	5/9	2.59	4.7	36	3/9	1.30	1.6
12	8/9	7.03	7.3	37	4/9	1.88	4.2
13	9/9	6.0	14.6	38	9/9	8.7	3.4
14	7/9	4.81	<0.3	39	1/9	0.38	<0.3
15	9/9	9.3	3.4	40	4/9	1.88	0.8
16	8/9	7.03	7.5	41	6/9	3.52	2.9
17	5/9	2.59	2.3	42	3/9	1.30	<0.3
18	9/9	13.4	4.2	43	0/9	<0.38	<0.3
19	6/9	3.52	3.4	44	0/9	<0.38	<0.3
20	0/9	<0.38	<0.3	45	3/9	1.30	0.5[3]
21	0/9	<0.38	<0.3	46	9/9	160.4	41.9
22	3/9	1.30	1.0	47	4/9	1.88	0.8
23	2/9	0.80	<0.3	48	9/9	17.4	7.8
24	1/9	0.38	<0.3	49	6/9	3.52	<0.3
25	3/9	1.30	<0.3	50	2/9	0.80	0.5

1 Number of replicates showing detection of HIV-1 RNA
2 4.2 mL assayed
3 Internal control failure
